# Decision-analytic models in the economic evaluation of community health worker programmes globally: a systematic review

**DOI:** 10.1136/bmjgh-2025-023076

**Published:** 2026-06-22

**Authors:** Siying Chen, Amrit Banstola, Cornelia Junghans Minton, Matthew Harris, Nana Anokye

**Affiliations:** 1 Department of Primary Care and Public Health, Imperial College London, London, UK; 2 Department of Health Sciences, Brunel University London, Uxbridge, UK; 3 Geller Institute for Ageing and Memory, University of West London, London, UK; 4 Brunel University London, Uxbridge, UK

**Keywords:** Decision Making, Delivery of Health Care, Global Health, Health Personnel, Health economics

## Abstract

**Introduction:**

Economic evidence on community health worker (CHW) programmes is crucial for scaling these initiatives. Although decision-analytic models (DAMs) are essential for projecting long-term value, it is unclear how rigorously they have been applied to CHW evaluations, potentially compromising the reliability and comparability of cost-effectiveness estimates used for policy decisions.

**Methods:**

A systematic review was conducted to identify full economic evaluations of CHW-led or CHW-integrated interventions that employed a DAM. Following Preferred Reporting Items for Systematic Reviews and Meta-Analyses (PRISMA) guidelines, six databases (Medline, Embase, Global Health, CINAHL, Web of Science and Scopus) were searched from inception to June 2025. Eligible studies were full economic evaluations assessing CHW-led or CHW-integrated interventions using DAMs. Study selection and data extraction were conducted independently by two reviewers. Methodological quality was appraised using the Philips checklist, and data were extracted on model type, data sources and validation practices. Findings were synthesised narratively across model structures, income groups and quality domains.

**Results:**

37 studies met the inclusion criteria. Decision trees were used in 32% of studies and Markov models in 30% with the remainder applying microsimulation, dynamic transmission or hybrid approaches. Most evaluations were undertaken in low- and middle-income countries, with few from low-income or high-income settings. Data constraints in low-income settings limited model complexity, whereas models in high-income settings tended to adopt more sophisticated structures but narrower intervention scopes. The mean quality score was 67%, with substantial gaps in model validation and limited exploration of structural uncertainty. Overall, 84% of studies concluded that CHW-led interventions were cost-effective, with incremental cost-effectiveness ratios generally favourable across settings.

**Conclusions:**

Although CHW interventions are generally cost-effective, the strength of this evidence is constrained by methodological limitations in existing models. Future modelling should prioritise rigorous validation, localisation of input data and explicit valuation of CHW and societal contributions to enhance the credibility of economic evidence for policy use.

**PROSPERO registration number:**

CRD420251066586.

**WHAT IS ALREADY KNOWN ON THIS TOPIC**

- Community health workers (CHW) programmes are widely recognised as an equitable and cost-effective approach to delivering primary healthcare and advancing universal health coverage, particularly in underserved areas.
- Decision-analytic models (DAMs) are well-established tools in health economics for predicting long-term costs and outcomes that go beyond the scope of individual trials.
- However, there has been no systematic review assessing how DAMs have been applied, assessed and reported in the economic evaluation of CHW programmes, leading to a notable gap in the evidence.

**WHAT THIS STUDY ADDS**

- We identify the predominance of decision tree models in low-income settings and Markov models in middle- and high-income contexts of CHW interventions.
- The methodological quality was moderate, with rare model validation and shallow uncertainty analyses.
- The sourcing of parameters showed a strong reliance on global datasets and published evidence, with low- and middle-income country studies frequently using expert opinion for costs.

**HOW THIS STUDY MIGHT AFFECT RESEARCH, PRACTICE OR POLICY**

- Further research is required to enhance the methodological rigour and contextual relevance of DAMs used in CHW programmes.
- Future evaluations should standardise model validation, uncertainty analysis and data reporting to improve comparability across settings.
- Policymakers and implementers should invest in robust local data systems and systematically evaluate the contributions of CHWs to ensure that economic evidence meaningfully informs equitable and sustainable health workforce policies.

## Introduction

 Health systems worldwide are facing a dual crisis: over 450 million people lack access to essential healthcare services, and more than one billion experience financial hardship due to healthcare costs.^1^ A global shortage of healthcare workers is exacerbating this crisis and impeding progress towards universal health coverage (UHC).^2^ The WHO identifies primary healthcare (PHC) as the most cost-effective and equitable pathway to achieving UHC.^2 3^

A cornerstone of this approach is the deployment of community health workers (CHWs), who are trusted community members with basic health training providing culturally appropriate health education, home visits, case identification, disease surveillance, treatment support, referrals and basic preventive care.^4 5^ CHWs reduce reliance on costly facility-based services, improve access in remote or marginalised areas, and strengthen the relationship between communities and formal health systems.^5^,^11^ The global relevance of CHWs is evident in their adoption across diverse contexts. Countries such as Brazil, Indonesia and Ghana have incorporated CHWs into national primary care strategies, while new initiatives in high-income settings, such as the Community Health and Wellbeing Workers programme in the UK, demonstrate the adaptability of CHW models.^12 13^ However, implementation remains uneven, and evidence on their long-term system-level impact is scarce.^11^

Despite extensive evidence that CHWs prevent an estimated 2.3 million maternal and child deaths annually and generate returns up to 16 times the initial investment, global investment in CHW programmes remains limited.^2 14 15^ The WHO estimates that an additional 1% of gross domestic product (GDP) is required to strengthen PHC and achieve UHC,^15^ yet only 2.5% of official development assistance has been directed specifically towards CHWs in the past decade, reflecting their continued underprioritisation despite their proven contributions to UHC and the Sustainable Development Goals.^15^ As investment in CHW programmes expands globally and across diverse settings, understanding their economic value has become increasingly important.

Economic evaluation provides a structured approach to comparing the costs and outcomes of CHW programmes with the potential uses of alternative resources.^16 17^ By their nature, CHW interventions are complex and frequently produce long-term system-level outcomes such as reduced hospitalisations, improved chronic disease management and greater equity, which extend beyond the scope of most primary studies.^7 18^ However, conventional trial-based evaluations often fail to capture these effects.^16^ Decision-analytic models (DAMs), such as decision trees, Markov models and microsimulations, are particularly well suited to addressing these challenges, as they can simulate long-term outcomes, integrate diverse data sources and systematically explore uncertainty through sensitivity and scenario analyses.^16 19^

To date, no systematic review has examined how DAMs have been used in the economic evaluation of CHW programmes. Recent evidence shows that existing economic evaluations of CHW interventions lack formal validation, rely on inconsistent outcome metrics and rarely incorporate context-specific mechanisms, which highlights the need for more rigorous and context-sensitive DAM.^20^

This systematic review therefore aims to synthesise and critically appraise existing evidence on the use of DAMs in CHW-related economic evaluations. Specifically, it seeks to:

1. Examine the data sources used to inform key model parameters (eg, costs, effectiveness and utilities) and assess how these choices vary across settings.
2. Identify the types of DAMs used for the economic evaluation of CHW programmes in different countries and health systems.
3. Evaluate the strengths and limitations of these models’ methodology to inform improvements to future economic evaluations.

## Methods

This systematic review was conducted and reported in accordance with the Preferred Reporting Items for Systematic Reviews and Meta-Analyses (PRISMA 2020) guidelines.^21^ The review protocol was prospectively registered on PROSPERO (registration number: CRD420251066586).

### Eligibility criteria

We developed eligibility criteria based on the PICO (Population–Intervention–Comparator–Outcome) framework. The population comprised studies evaluating CHW programmes or CHW-led interventions within any health system. This included studies that referred to CHWs using synonymous terms such as ‘community health volunteers’, ‘lay health workers’ or ‘health promoters’. The intervention encompassed activities involving health promotion, disease prevention, diagnosis, treatment support or care coordination, which were delivered or supported by CHWs. The comparator included usual care, standard facility-based care, no intervention or alternative provider models that did not involve CHWs (eg, nurse-led or physician-led care). Eligible outcomes were modelled economic results such as incremental cost-effectiveness ratios (ICERs), net monetary benefits (NMBs), cost–benefit ratios and other cost-effectiveness metrics.

We included full economic evaluations employing a DAM, such as a Markov model, decision tree, individual-level microsimulation or discrete event simulation. Examples of these models include cost-effectiveness (CEA), cost–utility (CUA) and cost–benefit (CBA) analyses.

Studies that reported only costs or only effects, rather than conducting a full economic evaluation, were excluded, as were reviews, case reports, protocols, conference abstracts, preprints and unpublished studies. Cost–minimisation and cost–consequence analyses were also excluded, since they do not generate the summary measure (eg, ICER or NMB) required for comparing interventions.

### Search strategy

Six databases were searched: MEDLINE, EMBASE, CINAHL Plus, Scopus, Web of Science and Global Health. All of the databases were searched from their inception dates to 16 June 2025, to maximise coverage of the available evidence. Only articles written in English were included. The reference lists of the included articles were also screened to identify any additional studies.

The search strategy combined controlled vocabulary and free-text terms across three categories: DAMs (eg, Markov models, microsimulation and decision trees); economic evaluations (eg, CEA, CUA and CBAs) and CHWs (eg, CHWs, lay health workers and village health workers). Boolean, truncation and adjacency operators were used to optimise sensitivity and precision.

The Polyglot Search Translator tool was used to translate the search strings across the databases.^22^ All outputs were then manually refined to ensure the subject headings and operators were mapped accurately. The full search strategies for each database are available in online supplemental appendix 1.

### Selection process

All records were imported into Covidence for de-duplication and screening.^23^ Two reviewers (SC and AB) independently screened the titles and abstracts of articles against the eligibility criteria, followed by a full-text assessment of those that were potentially eligible. Any disagreements between the reviewers were resolved by consensus.

### Data extraction and data items

A structured data extraction form was developed in Microsoft Excel based on the PRISMA 2020 checklist and the review’s objectives.^21^ The form was piloted on a sample of studies to ensure clarity and consistency. SC extracted the data and AB verified all the entries independently. Any disagreements between the reviewers were resolved by consensus.

The extracted data were organised into the following categories:

- General study characteristics: author(s), publication year, country/region, income classification, study title, objective and target population.
- CHW intervention details: cadre designation, health condition/disease area addressed.
- Economic evaluation findings and characteristics: results (including costs and outcomes as reported, with reported currency and price year where available), model type, software used, economic evaluation type (CEA, CUA, CBA), analytical perspective, time horizon.
- Model structure and parameters: description of the model, data sources for effectiveness and costs, discount rates and assumptions.
- Outputs and analysis: validation (internal, external or not reported), sensitivity analysis approach (deterministic, probabilistic or scenario-based), and transparency reporting.
- Authors’ reflections: conclusions, limitations and suggested future research directions.

The full data extraction template is provided in online supplemental appendix 2.

### Risk of bias and quality assessment

Two reviewers (SC and AB) independently assessed the methodological quality of each included study using the Philips checklist, as this remains the only comprehensive framework designed specifically for appraising DAM studies within economic evaluations.^24^ Each checklist item was scored as follows: Yes=1, No=0 and Not applicable=excluded. The overall score for each study was expressed as a percentage of ‘Yes’ responses out of all applicable items, enabling comparison across studies. Any disagreements were resolved by consensus. The quality appraisal informed the interpretation, but did not determine inclusion. Reporting biases were not formally assessed because no quantitative synthesis was planned.

### Data analysis and synthesis

Due to substantial heterogeneity in model structures, health conditions and data inputs, a meta-analysis was not feasible. Instead, a narrative synthesis approach was adopted to summarise and critically evaluate the existing evidence.

Data synthesis involved combining factual reporting with descriptive and thematic analysis in order to address the review objectives. Objective variables (eg, model type and CHW designation) were extracted as presented in the original studies. For economic results, extracted cost values were standardised to 2024 US dollars (US$) for comparability by applying GDP deflator indices and purchasing power parity conversion rates using the Campbell and Cochrane Economics Methods Group–EPPI-Centre Cost Converter (version 1.7).^25^ Where a price year was not reported, it was inferred from the costing period or source year (midpoint year used for multiyear periods). In contrast, interpretive variables (eg, methodological strengths and limitations, and data sources) were synthesised narratively. Countries were categorised by income group using the World Bank’s Country and Lending Groups classification for the 2026 fiscal year (FY26) to provide a consistent reference point across included studies.^26^

The methodological strengths were appraised against the following three indicators of good modelling practice: (1) explicit specification of model assumptions (eg, disease progression, intervention coverage, adherence and effectiveness); (2) evidence of model validation (internal, external or both) and (3) use of sensitivity analysis (deterministic, probabilistic or scenario-based). These indicators captured transparency, robustness and reproducibility, thus aligning with established standards for good modelling practice.^27^

The limitations reported by the study authors were extracted and categorised into seven domains: data limitations; limitations in model assumptions and structure; methodological uncertainty; uncertainty that was not fully accounted for; model dependency on calibration; limitations in effectiveness and measurement and limitations in study design. Brief category descriptions and illustrative examples are presented in table 3.

The parameter sources were synthesised into customised domains, including ‘Health Outcome Parameters’ and ‘Effectiveness and Epidemiological Inputs’. Subcategories were then used within each domain to ensure terminological consistency and enable cross-study comparison.

No data imputation was performed. The results were presented in structured summary tables to enable cross-study comparison.

## Results

**Figure 1:**
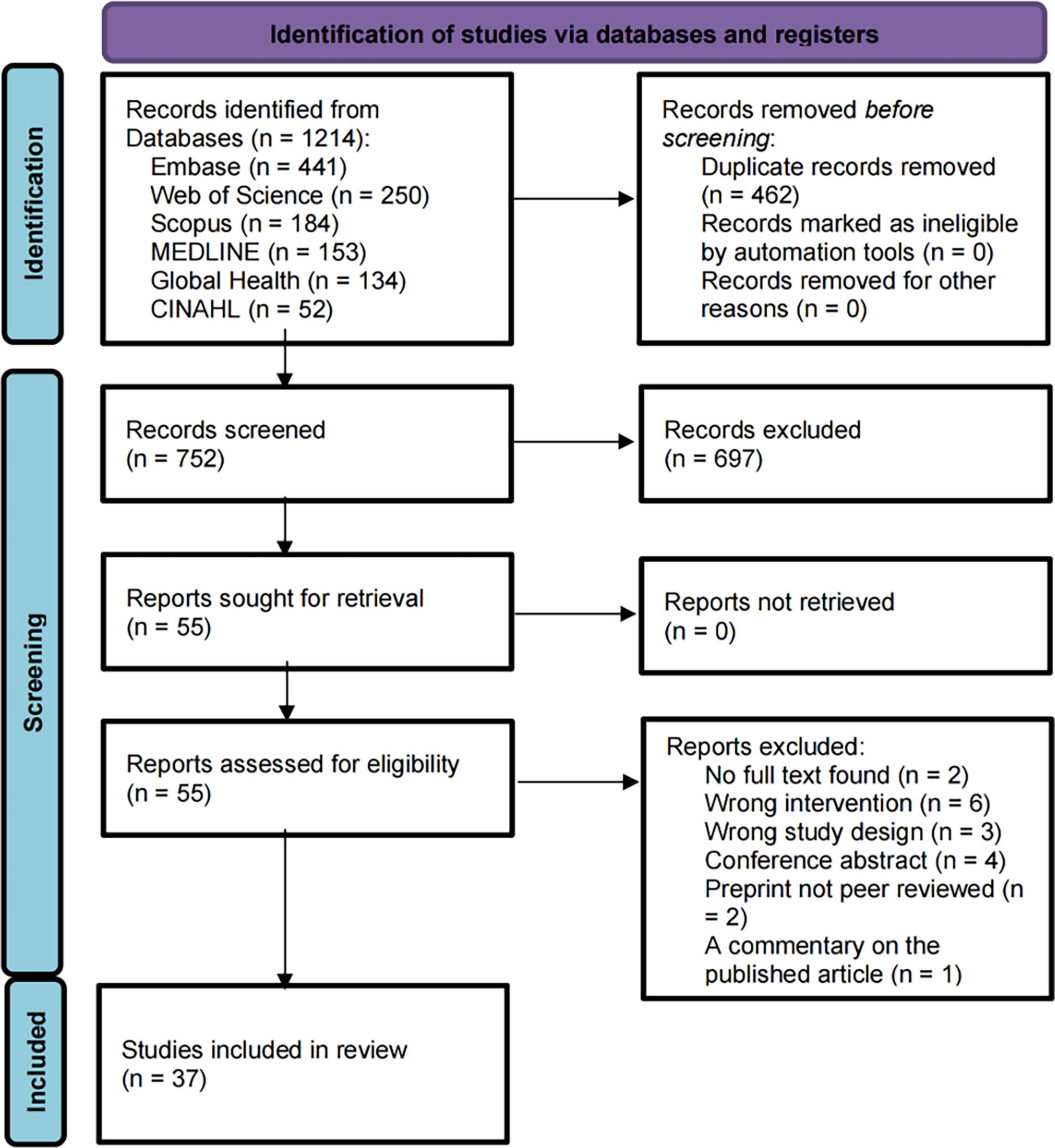
PRISMA flow diagram showing the selection process of studies included in the scoping review. PRISMA, Preferred Reporting Items for Systematic Reviews and Meta-Analyses.

A total of 1214 records were identified through database searches. After removing duplicates, 752 unique records were screened, of which 55 full-text articles were assessed for eligibility and 37 studies met the inclusion criteria (see figure 1).

A comprehensive overview of the characteristics and economic findings of the included studies is presented in online supplemental appendix 3.

The studies were published between 2012 and 2025, with nearly half appearing in 2021 or later,^28^,^44^ and covered 24 different countries in Asia, Africa, the Americas and Europe. India,^45^,^48^ Kenya,^3649^,^51^ South Africa^4452^,^54^ and the USA^55^,^58^ each contributed four studies, while Uganda^38 41 59^ contributed three. Most were single-country analyses (n=34). Based on the World Bank’s Country and Lending Groups classification for the 2026 fiscal year, these were primarily from middle-income economies (n=23), including 14 from lower-middle-income settings^2832 33 35 36 41 42 46^,^51 60^ and nine from upper-middle-income settings.^2934 37 44 52^,^54 61 62^ These were followed by studies from low-income settings (n=5)^38 40 41 43 59^ and high-income settings.^55^,^5863^ Three studies adopted a multicountry perspective, combining low-income and lower-middle-income settings.^31 39 64^

The most frequently evaluated health conditions were non-communicable diseases (n=11)^33 36 40 43 45 49 52 53 55 56 61^ and maternal, newborn and child health (MNCH) (n=8).^29 30 34 35 42 48 57 58^ Other topics included communicable diseases (n=6),^31 41 46 47 60 62^ MNCH and communicable diseases combined (n=7),^3237^,^39 44 50 64^ and a small number focused on cancer,^51 63^ nutrition-related conditions^28 59^ or health behaviours.^54^

The majority of the included studies were CEAs (n=22),^2830 31 35 36 38^,^45 47 48 50 51 59^ followed by CUAs (n=10).^2933 34 37 46 49 53^,^55 57^ Only one study conducted a CBA alone.^49^ Additionally, two studies applied both CEA and CUA methods^52 56^; one combined CEA with CBA^32^; and another combined CUA with a social return on investment framework.^54^

The target populations that were most commonly studied were children under five (n=8)^3238^,^41 43 45 49^ and pregnant women (n=8),^30 31 35 36 42 46 48 50^ followed by adults with chronic conditions, high-risk groups and marginalised communities. 24 studies used usual care as the comparator,^2829 31^,^37 39 40 42 43 45 46 48 52^ while the remaining studies compared CHW interventions against specific alternative strategies.

A total of 32 studies reported ICER-based outcomes. Of these, eight presented QALY-based ICERs ranging from full dominance to US$19 730 per QALY, with most estimates falling below US$2500 per QALY and a notable cluster around US$670–US$812 per QALY.^3334 37 53^,^57^ 16 studies reported DALY-based ICERs, spanning from US$6.58 to US$5242 per DALY, typically from US$173 to US$1100 per DALY^2829 32 35 36 39 45 46 48^,^52 64^ with two cases of complete dominance also observed.^43 62^ Eight studies presented highly variable estimates including life years gained, years of life lost (YLL), years of life saved (YLS) and case-based ICERs.^3031 40 41 44 59^,^61^ Four studies presented economic outcomes using alternative metrics, such as cost-effectiveness ratios, NMB, cost savings, financial protection and cost–benefit ratios.^38 42 47 63^ Overall, 31 out of 37 studies concluded that CHW interventions were cost-effective, whereas six studies produced context-dependent results.^31 32 34 38 60 62^

### Results of individual sources of evidence

Building on the descriptive overview of the included studies, the following sections present the results in relation to the three guiding questions of the review: (1) the data sources for the key parameters; (2) the modelling techniques used and (3) the strengths and limitations of the models’ methodology.

#### Data sources for key parameters

Across the 37 included studies, the sourcing of parameters in CHW economic evaluations varied according to data type and country context. Within the health outcomes domain, disability weights were primarily sourced from the Global Burden of Disease (GBD) study. Meanwhile, utility values and life expectancy or YLL inputs were generally obtained from published literature and standard life tables. Only a few studies applied a hybrid GBD and local calibration approach to improve contextual accuracy.

In the effectiveness and epidemiological domains, intervention effectiveness was primarily informed by randomised controlled trials (RCT), followed by published evidence and programme data. Disease progression, transition probabilities and mortality rates were largely derived from published sources or national statistics. Nationally representative household surveys were commonly used to estimate epidemiological and coverage parameters.

For cost inputs, local costing studies (n=24) and published literature (n=23) were the most frequent sources, particularly in middle-income settings. Cost databases (n=14) and policy documents (n=13) were also widely used. The distribution of cost data sources by country income group issummarised in table 1. 11 low- and middle-income country (LMIC) studies relied on expert opinion or assumptions, whereas none of the high-income country (HIC) studies did so. Six studies used household or national surveys to inform cost parameters.

**Table 1:** Distribution of cost data sources by country income group among included studies

	Total	High-income economies	Middle-income economies	Low-income economies	Temporary status of unclassification^26^
Local costing studies	24	2	16	5	1
Published literature	23	2	16	4	1
Cost databases	14	2	9	3	
Policy documents/guidelines	13	2	6	4	1
Programmatic/administrative data	11	2	6	3	
Expert opinion and assumptions	11	0	8	2	1
Household or national surveys	6	1	5	0	

Note: The values represent the number of studies included (n=37) that reported on each type of cost data source. As some studies used multiple sources, categories are not mutually exclusive. Country income groups were assigned according to the World Bank’s Country and Lending Groups classification for the 2026 fiscal year. ‘Temporary status of unclassification’ refers to Ethiopia’s current World Bank classification status for FY26.^26^

#### Modelling techniques used

Table 2 summarises the distribution of DAMs used in CHW economic evaluations across income levels and time horizons. Decision trees (n=12, 32%)^31 32 36 38 39 41 46 48 49 60 62 64^ were most prevalent in low- and lower-middle-income settings, being absent from HICs. In these contexts, decision tree models were typically applied to short, well-defined clinical pathways. However, half of these models adopted time horizons of 5 years or longer.^32 38 39 48 49 62^ Conversely, Markov models (n=11, 30%)^29 34 37 42 43 45 47 52 54 56 57^ were more prevalent in middle- and high-income economies.

**Table 2:** Types of decision-analytic models used in CHW economic evaluations by country income group and time horizon

	Count	%	Economic classification				Time horizon	
			Low-income	Lower-middle income	Upper-middle income	High-income	<2 years	5 years–lifetime
Decision tree	12	32	4	7	1	/	6	6
Markov model	11	30	1	3	5	2	1	10
Microsimulation/dynamic models	9	24	1	3	3	2	3	6
Custom/hybrid/other	5	14	1	2	/	2	2	3
Total	37	100%	7	15	9	5	12	25

Note: The values in the table represent the number of studies that used each model type. Percentages indicate the proportion of studies (n=37) that employed each model. The columns under ‘low-income’, ‘lower-middle income’, ‘upper-middle income’ and ‘high-income’ show the distribution of studies conducted in countries classified according to the World Bank’s Country and Lending Groups classification for the 2026 fiscal year.^26^ Studies conducted in a country with a temporary unclassified status for FY26^26 30^ are not assigned to any income group.

CHW, community health worker.

Microsimulation and dynamic models accounted for 24% of the studies, which were mainly from middle-income countries. Custom or hybrid models (11%) were identified across various health domains, integrating short- and long-term components, or linking economic outcomes with behavioural and social impacts. Detailed model specifications for these studies can be found in the online supplemental appendix 4.

The time horizons of the studies ranged from under 2 years to a lifetime. Around 70% of the studies simulated periods of between five and a lifetime.

#### Methodological strengths and limitations

Most studies demonstrated high transparency, with 92% explicitly reporting at least one modelling assumption.

Model validation emerged as a particularly weak methodological area. Only 16% of studies reported internal validation, 5% reported external validation and just 11% reported both. None of the decision tree models reported any validation. Among Markov models, validation was documented in just 3 out of 11 studies (27%), whereas almost all microsimulation and dynamic models underwent at least one validation procedure. Of the custom or hybrid models, only the Markov–decision tree hybrid underwent any form of validation.

Sensitivity analysis was conducted in 97% of studies, but the methodological depth varied considerably. The most common type of analysis was one-way deterministic sensitivity analysis (57%), followed by probabilistic sensitivity analysis (PSA) (51%), scenario analysis (41%), multiparameter analysis (24%) and multiway deterministic analysis (14%).

The most frequently reported methodological constraints were model assumptions and structural limitations (59%), followed by data limitations (54%) and methodological uncertainty (32%). Less common were limitations in the study design (27%), limitations in effectiveness and measurement (14%), and issues such as uncertainty not being fully accounted for (8%) or the model being dependent on calibration (3%) (see table 3).

**Table 3:** Thematic categories of self-reported methodological limitations in included studies (n=37)

Limitation category	Brief description	Example wording	N	% (n/37)
Model assumptions and structural limitations	Overly simplified or unrealistic model structure and assumptions	‘simplifying assumptions’; ‘does not include herd immunity’; ‘held risk factors constant’	22	59
Data limitations	Incomplete, non-representative, or proxy data affecting input accuracy and model reliability	‘lack of local data’; ‘not nationally representative’; ‘borrowed from other countries’	20	54
Methodological uncertainty	Lack of consensus or robustness in methodological choices (eg, thresholds, model selection)	‘no consensus on threshold’; ‘different model choice could change results’	12	32
Study design limitations	Design flaws such as lack of control group, small sample size or non-randomisation	‘non-randomised’; ‘no control group’; ‘costing/effect data were collected retrospectively (trial not designed for economic evaluation)’	10	27
Effectiveness and measurement limitations	Issues with internal/external validity of effect estimates, proxy outcomes or measurement tools	‘trial may not translate to real-world’; ‘proxy measure of incidence’	5	14
Uncertainty not fully accounted	Limited sensitivity or probabilistic analysis to characterise parameter or model uncertainty.	‘model does not account for parameter uncertainty’; ‘only point estimates used in PSA’	3	8
Model dependency on calibration	Results heavily dependent on calibration to limited or context-specific empirical data	‘results heavily dependent on calibration’; ‘calibration against independent datasets … differences could influence results’	1	3

Note: Example wording is illustrative and summarises recurring phrasing across included studies. N indicates the number of studies mentioning each limitation. As studies could report more than one type of methodological limitation, the categories are not mutually exclusive and the percentages do not sum to 100%.

PSA, probabilistic sensitivity analysis.

Detailed reporting of model assumptions, validation and sensitivity analyses, as well as core methodological limitations, are presented by DAM type in online supplemental appendix 4.

### Quality assessment

The average Philips Checklist score across all included studies was 66.7% (range: 45.7%–81.1%), indicating that the methodological quality was generally moderate, with notable variation across studies.

Several structural limitations were observed. For example, only 43% of studies clearly specified the decision-maker or intended audience, and 57% evaluated all feasible comparators, with 49% justifying exclusion of alternatives. Furthermore, some studies scoring above the overall mean (66.7%) were rated ‘No’ on the critical structural item assessing whether structural assumptions were reasonable given the model objective, perspective and scope.^39 48 49^

Reporting of data inputs was generally inconsistent. No studies assessed data quality, such as evaluating the representativeness, reliability or transferability of input parameters. Around 21% of studies relied on expert opinion to estimate parameters, yet the methods used to elicit this information were rarely described. Only 10% of studies explicitly reported applying a half-cycle correction and 38% of studies justified assumptions about treatment-effect persistence. No study addressed all four principal forms of uncertainty and only three explained why certain types were omitted.^44 45 61^ The most frequently analysed type of uncertainty was parameter uncertainty (86%), followed by methodological uncertainty (24%), uncertainty due to heterogeneity (22%), and structural uncertainty (14%).

The assessment of model consistency and validation was reported inconsistently. Only 25% of studies provided clear evidence of internal validation, such as logical consistency, completeness of transitions or computational checks. Just under half of the studies (44%) made use of external calibration against independent datasets, usually to explain any differences seen. Furthermore, 67% of studies compared their models with previously published models for the same disease or methodological domain, explaining differences in structure or parameterisation.

See online supplemental material 2 for the detailed Quality Appraisal table.

## Discussion

This systematic review found that most CHW interventions evaluated using DAMs were cost-effective. The most commonly applied modelling approaches were decision tree and Markov models. However, the overall methodological quality was moderate, with limitations including insufficient validation and uncertainty analysis and the underuse of local data.

The pattern of parameter sourcing reflects the tension between achieving global comparability and ensuring local relevance. While the widespread use of standardised global datasets ensures methodological consistency, it limits contextual validity.^12^ Without local calibration, such inputs may fail to capture the influence of cultural norms, health system performance or social determinants on health utilities, factors that could systematically bias cost-effectiveness estimates.^12^ These concerns have equity implications, as global datasets often reflect the norms of HICs and may inadequately represent the realities of LMICs.^65^

Similarly, while RCTs offer strong internal validity, the idealised conditions under which they are implemented, such as highly trained CHWs or unusually adherent participants, may yield effect sizes that overstate real-world performance.^16 17 66^ Conversely, trials conducted in environments with limited resources may underestimate achievable outcomes because implementation is often constrained by shortages of essential resources, high caseloads, inconsistent supervision and supply-chain interruptions rather than the intrinsic effectiveness of the CHW intervention.^5 12^ These contextual barriers can suppress measured effect sizes, thereby failing to reflect the programme’s performance under adequately resourced conditions. Both scenarios can distort policy expectations and lead to the inefficient allocation of resources.

Although household surveys provide valuable population-level data, they often lack the temporal and geographical granularity required for precise parameterisation, particularly in regions with variable disease burdens.^67 68^ In LMICs, the heavy reliance on expert opinion and assumed cost parameters reflects ongoing structural weaknesses in data systems.^69^ This dependence increases parameter uncertainty and reduces generalisability, corroborating previous findings that cost data in LMIC economic evaluations are often fragmented or incomplete.^70^

The pattern of modelling approaches was largely shaped by the type and availability of data in different settings. In low-income settings, decision tree models were used more frequently, which may reflect their relative feasibility when data inputs for more complex structures are limited.^19^ However, reliance on decision trees for long-term projections may not fully align with evaluation objectives, potentially contributing to the underestimation of sustained benefits.^17 19^ This reliance on simpler models appears to be driven by data scarcity rather than methodological choice, creating a self-perpetuating cycle in which limited resources lead to continued use of simpler models.

In contrast, Markov and microsimulation models were more prevalent in middle-income and HICs, where stronger data infrastructure and analytical capacity were available, particularly in areas where chronic and communicable diseases coexist.^71^,^73^ However, the limited number of CHW economic evaluations in high-income contexts may reflect differing research priorities, weaker incentives to demonstrate cost-effectiveness in well-resourced systems or the narrower role of CHWs in these settings.^10 74^ Although hybrid models show promise in integrating clinical, behavioural and social factors, they remain underused in LMICs due to technical and institutional constraints.^17 33 63 75^

Furthermore, simplified model specifications sometimes omit key non-clinical components of CHW interventions, such as the valuation of their labour.^74^ A key conceptual gap in existing economic models of CHWs is the failure to value the workers themselves.^8 12^ Most studies treat CHW time as a fixed or negligible cost, with few accounting for the opportunity cost of volunteer labour.^12 74 76^ Recent commentary has highlighted that many economic analyses also omit programme-level inputs including supervision and training, which contributes to the systematic undervaluation of CHW contributions and reinforces the risk of distorted cost-effectiveness estimates.^76^ This potentially underestimates the true economic value of CHW contributions and can distort cost-effectiveness estimates, particularly in contexts where CHWs provide substantial unpaid services.

Although transparent reporting of assumptions was common, merely stating assumptions does not guarantee empirical credibility. Verifying whether parameters such as disease progression or intervention effects are realistic requires domain-specific knowledge and contextually relevant data.^27^ This weakness corresponds directly to the most frequently reported limitation, ‘model assumptions and structural limitations’, highlighting the need for transparent and well-founded assumptions.

The scarcity of model validation, particularly in decision tree and hybrid applications, likely reflects broader issues such as limited data availability, the absence of standardised protocols and resource constraints. Conversely, the stronger validation practices observed in microsimulation and dynamic models may be driven by regulatory expectations and the presence of Health Technology Assessment frameworks in high-income settings. Systematic internal and external validation should be the minimum expectation for policy-relevant modelling, particularly in data-rich environments such as the UK.^27^

The inconsistent application of uncertainty analyses further undermines confidence in the reported results. Overreliance on one-way deterministic methods restricts the ability to capture interaction effects and structural uncertainty, factors that are highly relevant to CHW programmes, given their context-dependent nature and variable implementation quality.^617^,^19 27^

Simplified or static model structures may also omit critical CHW impact domains, such as empowerment, health equity and the navigation of social services.^6 77^ These omissions may bias results by underestimating broader societal and long-term benefits.^18^ Combined with data limitations, these modelling constraints can systematically undervalue the full spectrum of CHW benefits, leading to distorted conclusions about cost-effectiveness and ultimately influencing policy priorities and resource allocation decisions.

Taken together, our quality appraisal findings have important implications for the credibility of reported cost-effectiveness results. Where models rely on simplified or weakly justified structures and provide only partial assessment of heterogeneity and uncertainty, ICERs may be less robust and more sensitive to modelling choices. Notably, while 59% of authors self-reported structural simplifications (see table 3), only 8% acknowledged inadequate characterisation of uncertainty. This suggests insufficient field-wide recognition of how this deficit undermines decision confidence, despite our appraisal finding this to be equally prevalent. Our appraisal also highlights that reporting quality and conceptual validity can diverge, reinforcing the need to align model structure more explicitly with the stated objective, perspective and scope.^27^ Conversely, some innovative approaches provide valuable insights despite modest checklist scores; for example, Tampi *et al* employed OpenMalaria, an open-source transmission-dynamic microsimulation platform, to capture time-varying infection dynamics and intervention effects beyond traditional cohort models^28^; agent-based approaches (eg, IndiaSim) similarly enable representation of heterogeneous individuals and interaction-driven processes.^47^

### Strengths and limitations

This review is the first to comprehensively synthesise modelling structures, parameter sources and validation practices. It offers insights to inform more rigorous and policy-relevant CHW economic evaluations.

However, several limitations should be noted. Despite an extensive search, only peer-reviewed English-language publications were included, which may have excluded relevant evidence from countries such as Brazil, where CHW studies are often published in Portuguese or in non-indexed sources. The search strategy may also have missed studies that were not explicitly labelled as economic or modelling analyses. While the Philips Checklist enabled a structured appraisal, it may not fully capture the unique modelling challenges of CHW contexts, particularly the difficulties of parameterising ‘soft’ inputs such as trust and health education, and of accounting for community-wide spillover effects beyond directly targeted patients. In addition, some interpretive judgement was required when classifying models and limitations due to inconsistent reporting. Finally, although the use of FY26 income classifications provided a consistent reference point across studies, countries may move between income groups over time, and their classification at the time of intervention delivery or cost-data generation may have differed from their FY26 classification.^26 78^ This should be considered when interpreting patterns by income group.

Despite these limitations, this review provides a thorough synthesis of current modelling practices, identifies key areas for future research and offers practical recommendations to improve the quality and contextual relevance of CHW economic evaluations.

### Implications for policy, practice and future research

To optimise the use of DAMs in evaluating CHW programmes, policymakers must strengthen both the methodological standards and the local data foundations that underpin modelled evidence. Transparent reporting and verification/validation (where feasible) should become standard practice, supported by national or institutional guidelines such as the International Society for Pharmacoeconomics and Outcomes Research - Society for Medical Decision Making Modelling Good Research Practices, to ensure consistency and comparability across settings.^27^ Investment in CHW-relevant local data systems is equally critical, including cost databases, national health surveys and monitoring systems that track programme coverage, intensity and effectiveness. Such efforts would align with the WHO’s recommendations to institutionalise the use of local data for planning and evaluation purposes.^79^ Where non-local inputs are used, transferability should be justified, and the models should be calibrated to local epidemiology and service-use patterns wherever possible. Finally, future economic evaluations should explicitly recognise the value of CHW time and their broader social contributions. Including the opportunity cost of volunteer labour in cost-effectiveness estimates would yield more accurate results and support the development of equitable and sustainable financing strategies for CHW programmes.

Across included studies, implementation fidelity and delivery constraints (eg, training intensity, supervision quality, workload, resource availability and supply-chain reliability) were rarely represented despite their likely impact on outcomes and costs. Strengthening routine programme data capture on these determinants would enable models to parameterise realistic implementation scenarios and better reflect real-world variation in CHW performance, improving usefulness for local planning.

Future research should ensure that modelling approaches are explicitly aligned with the evaluation objectives and CHW programme theory. To estimate long-term cost-effectiveness, state-transition models (including Markov) and/or individual-level microsimulation models are needed to better capture time-dependent processes, complex intervention pathways and individual heterogeneity,^16 19^ alongside transparent uncertainty analysis (eg, PSA) and implementation-relevant scenario/subgroup analyses. Despite the increasing prevalence of CHW-like roles in systems such as the UK’s primary care networks, evidence from HICs remains limited. Future research should explore how advanced modelling frameworks can be adapted to these contexts by leveraging the availability of detailed epidemiological and administrative data. From an implementation science perspective, decision models could also guide evidence generation by testing which implementation determinants drive value and decision uncertainty, helping prioritise data collection.^80^

## Conclusions

The use of DAMs to evaluate CHW programmes is increasing, but methodological rigour remains inconsistent, with most studies lacking validation and relying on proxy data. They also tend to often overlook the time and social contributions of CHWs. Strengthening methodological standards through improved transparency, dynamic modelling approaches such as Markov or microsimulation models, and locally grounded data will generate stronger, more equitable evidence to inform global health policy and investment decisions.

## Supplementary material

10.1136/bmjgh-2025-023076 online supplemental file 1

10.1136/bmjgh-2025-023076 online supplemental file 2

## Data Availability

Data are available on reasonable request.
