# Supplementary material for: Decision-analytic models in the economic evaluation of community health worker programmes globally: a systematic review
Source: BMJ Glob Health. 2026 Jun 22;11(6):e023076. doi: 10.1136/bmjgh-2025-023076 (PMC13288870; doi:10.1136/bmjgh-2025-023076)
Supplement: online supplemental file 1 [file bmjgh-11-6-s001.docx]

# Supplementary Materials

## Appendix 1. Search Strategy

Appendix Table 1. Full Ovid MEDLINE Search Strategy

| **#** | **Search Terms** |
| --- | --- |
| 1 | Markov chains/ |
| 2 | exp Computer Simulation/ |
| 3 | exp decision support techniques/ |
| 4 | monte carlo method/ |
| 5 | exp Decision Trees/ |
| 6 | Models, Theoretical/ |
| 7 | Models, Economic/ |
| 8 | ((decision* adj2 model*) or (decision-analytic* adj2 (model* or approach*)) or ((model* adj3 (approach* or study or simulat*)) and economic) or (simulation adj5 model*) or mathematical model* or (dynamic adj2 model*) or economic model*).tw. |
| 9 | ((markov adj5 (model* or framework* or chain*)) or state transition model*).tw. |
| 10 | ((decision* adj2 tree*) or (decision-making adj2 tree*) or tree model*).tw. |
| 11 | ((micro? adj2 simulation*) or microsimulation or (individual-level adj2 simulation*) or (patient-level adj2 simulation*)).tw. |
| 12 | (monte carlo or discrete event simulation).tw. |
| 13 | 1 or 2 or 3 or 4 or 5 or 6 or 7 or 8 or 9 or 10 or 11 or 12 |
| 14 | Economics/ |
| 15 | costs and cost analysis/ or cost-benefit analysis/ or "cost of illness"/ or health care costs/ or health expenditures/ |
| 16 | exp Resource Allocation/ |
| 17 | disability-adjusted life years/ or quality-adjusted life years/ |
| 18 | exp models, economic/ |
| 19 | ((economic* adj2 (evaluat* or analys* or assess* or model*)) or (cost adj2 analysis) or (cost adj2 evaluation)).tw. |
| 20 | ((cost adj2 effective*) or (cost-effectiveness adj2 (analys* or study or evaluat* or model* or decision*)) or CEA or ICER or (incremental cost adj2 effectiveness ratio)).tw. |
| 21 | ((cost adj2 utilit*) or (cost-utility adj2 (analys* or ratio* or impact)) or CUA or (QALY or QALYs or "quality-adjusted life year*")).tw. |
| 22 | ((cost adj2 benefit*) or (cost-benefit adj2 (analys* or study)) or (benefit adj2 cost adj2 evaluat*) or CBA).tw. |
| 23 | (economic feasib* or financial viabilit*).tw. |
| 24 | (((value adj2 money) or budget* or costly or costing or price* or expenditure* or (DALY or DALYs or "disability-adjusted life year*")).tw. or health resource.mp.) adj2 (allocat* or utiliz*).tw. |
| 25 | 14 or 15 or 16 or 17 or 18 or 19 or 20 or 21 or 22 or 23 or 24 |
| 26 | Community Health Workers/ |
| 27 | ("community health worker*" or "community-based health worker*" or "village health worker*" or "community health volunteer*" or "community health agent*" or "community care worker*" or "primary health care worker*").tw. |
| 28 | ((lay adj2 worker*) or ("volunteer health worker*" or "community volunteer*" or "outreach worker*" or "link worker*" or "linkworker*" or "health extension worker*" or "health promoter*" or "community health promoter*" or "community-based practitioner*" or "community case management worker*" or "close-to-community provider*" or "expert patient*" or "home health aide*" or "health auxiliar*" or "paraprofessional*" or "barefoot doctor*" or "frontline health worker*")).tw. |
| 29 | (((community or village) adj3 (health worker* or provider*)) or ((lay or volunteer* or outreach or support or link*) adj5 (worker* or aide* or provider* or practitioner*))).tw. |
| 30 | ((CHW or "community health worker") adj2 intervention*).tw. |
| 31 | 26 or 27 or 28 or 29 or 30 |
| 32 | 13 and 25 and 31 |

Appendix Table 2. Embase Classic+Embase Search Strategy

| **#** | **Search Terms** |
| --- | --- |
| 1 | exp Markov chain/ |
| 2 | exp computer simulation/ |
| 3 | exp decision support system/ |
| 4 | exp Monte Carlo method/ |
| 5 | exp "decision tree"/ |
| 6 | exp theoretical model/ |
| 7 | ((decision* adj2 model*) or (decision-analytic* adj2 (model* or approach*)) or ((model* adj3 (approach* or study or simulat*)) and economic) or (simulation adj5 model*) or "mathematical model*" or (dynamic adj2 model*) or "economic model*").tw. |
| 8 | ((markov adj5 (model* or framework* or chain*)) or "state transition model*").tw. |
| 9 | ((decision* adj2 tree*) or (decision-making adj2 tree*) or "tree model*").tw. |
| 10 | ((micro? adj2 simulation*) or microsimulation or (individual-level adj2 simulation*) or (patient-level adj2 simulation*)).tw. |
| 11 | ("monte carlo" or "discrete event simulation").tw. |
| 12 | 1 or 2 or 3 or 4 or 5 or 6 or 7 or 8 or 9 or 10 or 11 |
| 13 | exp health economics/ |
| 14 | cost benefit analysis/ |
| 15 | exp "cost effectiveness analysis"/ |
| 16 | exp "health care cost"/ |
| 17 | exp economic model/ |
| 18 | exp econometric model/ |
| 19 | exp quality adjusted life year/ |
| 20 | exp resource allocation/ |
| 21 | ((economic* adj2 (evaluat* or analys* or assess* or model*)) or (cost adj2 analysis) or (cost adj2 evaluation)).tw. |
| 22 | ((cost adj2 effective*) or (cost-effectiveness adj2 (analys* or study or evaluat* or model* or decision*)) or CEA or ICER or ("incremental cost" adj2 "effectiveness ratio")).tw. |
| 23 | ((cost adj2 utilit*) or (cost-utility adj2 (analys* or ratio* or impact)) or CUA or (QALY or QALYs or "quality-adjusted life year*")).tw. |
| 24 | ((cost adj2 benefit*) or (cost-benefit adj2 (analys* or study)) or (benefit adj2 cost adj2 evaluat*) or CBA).tw. |
| 25 | ("economic feasib*" or "financial viabilit*").tw. |
| 26 | (((value adj2 money) or budget* or costly or costing or price* or expenditure* or (DALY or DALYs or "disability-adjusted life year*")).tw. or "health resource".mp.) adj2 (allocat* or utiliz*).tw. |
| 27 | 13 or 14 or 15 or 16 or 17 or 18 or 19 or 20 or 21 or 22 or 23 or 24 or 25 or 26 |
| 28 | exp health auxiliary/ |
| 29 | ("community health worker*" or "community-based health worker*" or "village health worker*" or "community health volunteer*" or "community health agent*" or "community care worker*" or "primary health care worker*").tw. |
| 30 | ((lay adj2 worker*) or ("volunteer health worker*" or "community volunteer*" or "outreach worker*" or "link worker*" or linkworker* or "health extension worker*" or "health promoter*" or "community health promoter*" or "community-based practitioner*" or "community case management worker*" or "close-to-community provider*" or "expert patient*" or "home health aide*" or "health auxiliar*" or paraprofessional* or "barefoot doctor*" or "frontline health worker*")).tw. |
| 31 | (((community or village) adj3 ("health worker*" or provider*)) or ((lay or volunteer* or outreach or support or link*) adj5 (worker* or aide* or provider* or practitioner*))).tw. |
| 32 | ((CHW or "community health worker") adj2 intervention*).tw. |
| 33 | 28 or 29 or 30 or 31 or 32 |
| 34 | 12 and 27 and 33 |

Appendix Table 3. CINAHL Plus Search Strategy

| **#** | **Search Terms** |
| --- | --- |
| S1 | (MH "Decision Support Techniques+") OR (MH "Statistics") |
| S2 | (MH "Decision Support Systems, Clinical") OR (MH "Decision Trees+") |
| S3 | (MH "Computer Simulation") |
| S4 | (MH "Hidden Markov Models") |
| S5 | (((TI decision* OR AB decision*) N1 (TI model* OR AB model*)) OR ((TI decision-analytic* OR AB decision-analytic*) N1 ((TI model* OR AB model*) OR (TI approach* OR AB approach*))) OR (((TI model* OR AB model*) N2 ((TI approach* OR AB approach*) OR (TI study OR AB study) OR (TI simulat* OR AB simulat*))) AND (TI economic OR AB economic)) OR ((TI simulation OR AB simulation) N4 (TI model* OR AB model*)) OR (TI "mathematical model*" OR AB "mathematical model*") OR ((TI dynamic OR AB dynamic) N1 (TI model* OR AB model*)) OR (TI "economic model*" OR AB "economic model*")) |
| S6 | (((TI markov OR AB markov) N4 ((TI model* OR AB model*) OR (TI framework* OR AB framework*) OR (TI chain* OR AB chain*))) OR (TI "state transition model*" OR AB "state transition model*")) |
| S7 | (((TI decision* OR AB decision*) N1 (TI tree* OR AB tree*)) OR ((TI decision-making OR AB decision-making) N1 (TI tree* OR AB tree*)) OR (TI "tree model*" OR AB "tree model*")) |
| S8 | (((TI micro# OR AB micro#) N1 (TI simulation* OR AB simulation*)) OR (TI microsimulation OR AB microsimulation) OR ((TI individual-level OR AB individual-level) N1 (TI simulation* OR AB simulation*)) OR ((TI patient-level OR AB patient-level) N1 (TI simulation* OR AB simulation*))) |
| S9 | ((TI "monte carlo" OR AB "monte carlo") OR (TI "discrete event simulation" OR AB "discrete event simulation")) |
| S10 | S1 OR S2 OR S3 OR S4 OR S5 OR S6 OR S7 OR S8 OR S9 |
| S11 | (MH "Economic Aspects of Illness") OR (MH "Economics") |
| S12 | (MH "Costs and Cost Analysis") OR (MH "Cost Effectiveness Analysis") OR (MH "Cost Benefit Analysis") OR (MH "Health Care Costs") OR (MH "Health Expenditures") |
| S13 | (MH "Resource Allocation+") |
| S14 | (MH "Quality-Adjusted Life Years") OR (MH "Disability-Adjusted Life Years") |
| S15 | (((TI economic* OR AB economic*) N1 ((TI evaluat* OR AB evaluat*) OR (TI analys* OR AB analys*) OR (TI assess* OR AB assess*) OR (TI model* OR AB model*))) OR ((TI cost OR AB cost) N1 (TI analysis OR AB analysis)) OR ((TI cost OR AB cost) N1 (TI evaluation OR AB evaluation))) |
| S16 | (((TI cost OR AB cost) N1 (TI effective* OR AB effective*)) OR ((TI cost-effectiveness OR AB cost-effectiveness) N1 ((TI analys* OR AB analys*) OR (TI study OR AB study) OR (TI evaluat* OR AB evaluat*) OR (TI model* OR AB model*) OR (TI decision* OR AB decision*))) OR (TI CEA OR AB CEA) OR (TI ICER OR AB ICER) OR ((TI "incremental cost" OR AB "incremental cost") N1 (TI "effectiveness ratio" OR AB "effectiveness ratio"))) |
| S17 | (((TI cost OR AB cost) N1 (TI utilit* OR AB utilit*)) OR ((TI cost-utility OR AB cost-utility) N1 ((TI analys* OR AB analys*) OR (TI ratio* OR AB ratio*) OR (TI impact OR AB impact))) OR (TI CUA OR AB CUA) OR ((TI QALY OR AB QALY) OR (TI QALYs OR AB QALYs) OR (TI "quality-adjusted life year*" OR AB "quality-adjusted life year*"))) |
| S18 | (((TI cost OR AB cost) N1 (TI benefit* OR AB benefit*)) OR ((TI cost-benefit OR AB cost-benefit) N1 ((TI analys* OR AB analys*) OR (TI study OR AB study))) OR ((TI benefit OR AB benefit) N1 (TI cost OR AB cost) N1 (TI evaluat* OR AB evaluat*)) OR (TI CBA OR AB CBA)) |
| S19 | ((TI "economic feasib*" OR AB "economic feasib*") OR (TI "financial viabilit*" OR AB "financial viabilit*")) |
| S20 | ((((TI value OR AB value) N1 (TI money OR AB money)) OR (TI budget* OR AB budget*) OR (TI costly OR AB costly) OR (TI costing OR AB costing) OR (TI price* OR AB price*) OR (TI expenditure* OR AB expenditure*) OR ((TI DALY OR AB DALY) OR (TI DALYs OR AB DALYs) OR (TI "disability-adjusted life year*" OR AB "disability-adjusted life year*"))) OR "health resource") N1 ((TI allocat* OR AB allocat*) OR (TI utiliz* OR AB utiliz*)) |
| S21 | S11 OR S12 OR S13 OR S14 OR S15 OR S16 OR S17 OR S18 OR S19 OR S20 |
| S22 | (MH "Community Health Workers") |
| S23 | ((TI "community health worker*" OR AB "community health worker*") OR (TI "community-based health worker*" OR AB "community-based health worker*") OR (TI "village health worker*" OR AB "village health worker*") OR (TI "community health volunteer*" OR AB "community health volunteer*") OR (TI "community health agent*" OR AB "community health agent*") OR (TI "community care worker*" OR AB "community care worker*") OR (TI "primary health care worker*" OR AB "primary health care worker*")) |
| S24 | (((TI lay OR AB lay) N1 (TI worker* OR AB worker*)) OR ((TI "volunteer health worker*" OR AB "volunteer health worker*") OR (TI "community volunteer*" OR AB "community volunteer*") OR (TI "outreach worker*" OR AB "outreach worker*") OR (TI "link worker*" OR AB "link worker*") OR (TI linkworker* OR AB linkworker*) OR (TI "health extension worker*" OR AB "health extension worker*") OR (TI "health promoter*" OR AB "health promoter*") OR (TI "community health promoter*" OR AB "community health promoter*") OR (TI "community-based practitioner*" OR AB "community-based practitioner*") OR (TI "community case management worker*" OR AB "community case management worker*") OR (TI "close-to-community provider*" OR AB "close-to-community provider*") OR (TI "expert patient*" OR AB "expert patient*") OR (TI "home health aide*" OR AB "home health aide*") OR (TI "health auxiliar*" OR AB "health auxiliar*") OR (TI paraprofessional* OR AB paraprofessional*) OR (TI "barefoot doctor*" OR AB "barefoot doctor*") OR (TI "frontline health worker*" OR AB "frontline health worker*"))) |
| S25 | ((((TI community OR AB community) OR (TI village OR AB village)) N2 ((TI "health worker*" OR AB "health worker*") OR (TI provider* OR AB provider*))) OR (((TI lay OR AB lay) OR (TI volunteer* OR AB volunteer*) OR (TI outreach OR AB outreach) OR (TI support OR AB support) OR (TI link* OR AB link*)) N4 ((TI worker* OR AB worker*) OR (TI aide* OR AB aide*) OR (TI provider* OR AB provider*) OR (TI practitioner* OR AB practitioner*)))) |
| S26 | (((TI CHW OR AB CHW) OR (TI "community health worker" OR AB "community health worker")) N1 (TI intervention* OR AB intervention*)) |
| S27 | S22 OR S23 OR S24 OR S25 OR S26 |
| S28 | S10 AND S21 AND S27 |

Appendix Table 4. Scoups Search Strategy

| 1 | ( TITLE-ABS-KEY ( ( ( decision* W/1 model* ) OR ( decision-analytic* W/1 ( model* OR approach* ) ) OR ( ( model* W/2 ( approach* OR study OR simulat* ) ) AND economic ) OR ( simulation W/4 model* ) OR "mathematical model*" OR ( dynamic W/1 model* ) OR "economic model*" ) ) OR TITLE-ABS-KEY ( ( ( markov W/4 ( model* OR framework* OR chain* ) ) OR "state transition model*" ) ) OR TITLE-ABS-KEY ( ( ( decision* W/1 tree* ) OR ( decision-making W/1 tree* ) OR "tree model*" ) ) OR TITLE-ABS-KEY ( ( ( micro* W/1 simulation* ) OR microsimulation OR ( individual-level W/1 simulation* ) OR ( patient-level W/1 simulation* ) ) ) OR TITLE-ABS-KEY ( ( "monte carlo" OR "discrete event simulation" ) ) ) |
| --- | --- |
| 2 | ( TITLE-ABS-KEY ( ( economic* W/1 ( evaluat* OR analys* OR assess* OR model* ) ) OR ( cost W/1 analysis ) OR ( cost W/1 evaluation ) ) OR TITLE-ABS-KEY ( ( cost W/1 effective* ) OR ( cost-effectiveness W/1 ( analys* OR study OR evaluat* OR model* OR decision* ) ) OR cea OR icer OR ( "incremental cost" W/1 "effectiveness ratio" ) ) OR TITLE-ABS-KEY ( ( cost W/1 utilit* ) OR ( cost-utility W/1 ( analys* OR ratio* OR impact ) ) OR cua OR ( qaly OR qalys OR "quality-adjusted life year*" ) ) OR TITLE-ABS-KEY ( ( cost W/1 benefit* ) OR ( cost-benefit W/1 ( analys* OR study ) ) OR ( benefit W/1 cost W/1 evaluat* ) OR cba ) OR TITLE-ABS-KEY ( "economic feasib*" OR "financial viabilit*" ) OR TITLE-ABS-KEY ( ( ( value W/2 money ) OR budget* OR costly OR costing OR price* OR expenditure* OR ( daly OR dalys OR "disability-adjusted life year*" ) OR "health resource" ) AND ( allocat* OR utiliz* ) ) ) |
| 3 | ( TITLE-ABS-KEY ( ( "community health worker*" OR "community-based health worker*" OR "village health worker*" OR "community health volunteer*" OR "community health agent*" OR "community care worker*" OR "primary health care worker*" ) ) OR TITLE-ABS-KEY ( ( ( lay W/1 worker* ) OR ( "volunteer health worker*" OR "community volunteer*" OR "outreach worker*" OR "link worker*" OR linkworker* OR "health extension worker*" OR "health promoter*" OR "community health promoter*" OR "community-based practitioner*" OR "community case management worker*" OR "close-to-community provider*" OR "expert patient*" OR "home health aide*" OR "health auxiliar*" OR paraprofessional* OR "barefoot doctor*" OR "frontline health worker*" ) ) ) OR TITLE-ABS-KEY ( ( ( ( community OR village ) W/2 ( "health worker*" OR provider* ) ) OR ( ( lay OR volunteer* OR outreach OR support OR link* ) W/4 ( worker* OR aide* OR provider* OR practitioner* ) ) ) ) OR TITLE-ABS-KEY ( ( ( chw OR "community health worker" ) W/1 intervention* ) ) ) |
| 4 | ( ( TITLE-ABS-KEY ( ( "community health worker*" OR "community-based health worker*" OR "village health worker*" OR "community health volunteer*" OR "community health agent*" OR "community care worker*" OR "primary health care worker*" ) ) OR TITLE-ABS-KEY ( ( ( lay W/1 worker* ) OR ( "volunteer health worker*" OR "community volunteer*" OR "outreach worker*" OR "link worker*" OR linkworker* OR "health extension worker*" OR "health promoter*" OR "community health promoter*" OR "community-based practitioner*" OR "community case management worker*" OR "close-to-community provider*" OR "expert patient*" OR "home health aide*" OR "health auxiliar*" OR paraprofessional* OR "barefoot doctor*" OR "frontline health worker*" ) ) ) OR TITLE-ABS-KEY ( ( ( ( community OR village ) W/2 ( "health worker*" OR provider* ) ) OR ( ( lay OR volunteer* OR outreach OR support OR link* ) W/4 ( worker* OR aide* OR provider* OR practitioner* ) ) ) ) OR TITLE-ABS-KEY ( ( ( chw OR "community health worker" ) W/1 intervention* ) ) ) ) AND ( ( TITLE-ABS-KEY ( ( economic* W/1 ( evaluat* OR analys* OR assess* OR model* ) ) OR ( cost W/1 analysis ) OR ( cost W/1 evaluation ) ) OR TITLE-ABS-KEY ( ( cost W/1 effective* ) OR ( cost-effectiveness W/1 ( analys* OR study OR evaluat* OR model* OR decision* ) ) OR cea OR icer OR ( "incremental cost" W/1 "effectiveness ratio" ) ) OR TITLE-ABS-KEY ( ( cost W/1 utilit* ) OR ( cost-utility W/1 ( analys* OR ratio* OR impact ) ) OR cua OR ( qaly OR qalys OR "quality-adjusted life year*" ) ) OR TITLE-ABS-KEY ( ( cost W/1 benefit* ) OR ( cost-benefit W/1 ( analys* OR study ) ) OR ( benefit W/1 cost W/1 evaluat* ) OR cba ) OR TITLE-ABS-KEY ( "economic feasib*" OR "financial viabilit*" ) OR TITLE-ABS-KEY ( ( ( value W/2 money ) OR budget* OR costly OR costing OR price* OR expenditure* OR ( daly OR dalys OR "disability-adjusted life year*" ) OR "health resource" ) AND ( allocat* OR utiliz* ) ) ) ) AND ( ( TITLE-ABS-KEY ( ( ( decision* W/1 model* ) OR ( decision-analytic* W/1 ( model* OR approach* ) ) OR ( ( model* W/2 ( approach* OR study OR simulat* ) ) AND economic ) OR ( simulation W/4 model* ) OR "mathematical model*" OR ( dynamic W/1 model* ) OR "economic model*" ) ) OR TITLE-ABS-KEY ( ( ( markov W/4 ( model* OR framework* OR chain* ) ) OR "state transition model*" ) ) OR TITLE-ABS-KEY ( ( ( decision* W/1 tree* ) OR ( decision-making W/1 tree* ) OR "tree model*" ) ) OR TITLE-ABS-KEY ( ( ( micro* W/1 simulation* ) OR microsimulation OR ( individual-level W/1 simulation* ) OR ( patient-level W/1 simulation* ) ) ) OR TITLE-ABS-KEY ( ( "monte carlo" OR "discrete event simulation" ) ) ) ) |

Appendix Table 5. Web of Science Search Strategy

| **#** | **Search Terms** |
| --- | --- |
| 1 | (TS=(((decision* NEAR/1 model*) OR (decision-analytic* NEAR/1 (model* OR approach*)) OR ((model* NEAR/2 (approach* OR study OR simulat*)) AND economic) OR (simulation NEAR/4 model*) OR "mathematical model*" OR (dynamic NEAR/1 model*) OR "economic model*")) OR TS=(((markov NEAR/4 (model* OR framework* OR chain*)) OR "state transition model*")) OR TS=(((decision* NEAR/1 tree*) OR (decision-making NEAR/1 tree*) OR "tree model*")) OR TS=(((micro$ NEAR/1 simulation*) OR microstimulation OR (individual-level NEAR/1 simulation*) OR (patient-level NEAR/1 simulation*))) OR TS=(("monte carlo" OR "discrete event simulation"))) NOT (SILOID==("PPRN")) |
| 2 | TS=((economic* NEAR/1 (evaluat* OR analys* OR assess* OR model*)) OR (cost NEAR/1 analysis) OR (cost NEAR/1 evaluation)) OR TS=((cost NEAR/1 effective*) OR (cost-effectiveness NEAR/1 (analys* OR study OR evaluat* OR model* OR decision*)) OR CEA OR ICER OR ("incremental cost" NEAR/1 "effectiveness ratio")) OR TS=((cost NEAR/1 utilit*) OR (cost-utility NEAR/1 (analys* OR ratio* OR impact)) OR CUA OR QALY OR QALYs OR "quality-adjusted life year*") OR TS=((cost NEAR/1 benefit*) OR (cost-benefit NEAR/1 (analys* OR study)) OR (benefit NEAR/1 cost NEAR/1 evaluat*) OR CBA) OR TS=("economic feasib*" OR "financial viabilit*") OR TS=(((value NEAR/1 money) OR budget* OR costly OR costing OR price* OR expenditure* OR DALY OR DALYs OR "disability-adjusted life year*") NEAR/1 (allocat* OR utiliz*) OR ("health resource" NEAR/1 (allocat* OR utiliz*))) |
| 3 | ( TS=( ("community health worker*" OR "community-based health worker*" OR "village health worker*" OR "community health volunteer*" OR "community health agent*" OR "community care worker*" OR "primary health care worker*") OR ((lay NEAR/1 worker*) OR "volunteer health worker*" OR "community volunteer*" OR "outreach worker*" OR "link worker*" OR linkworker* OR "health extension worker*" OR "health promoter*" OR "community health promoter*" OR "community-based practitioner*" OR "community case management worker*" OR "close-to-community provider*" OR "expert patient*" OR "home health aide*" OR "health auxiliar*" OR paraprofessional* OR "barefoot doctor*" OR "frontline health worker*") OR (((community OR village) NEAR/2 ("health worker*" OR provider*)) OR ((lay OR volunteer* OR outreach OR support OR link*) NEAR/4 (worker* OR aide* OR provider* OR practitioner*))) OR ((CHW OR "community health worker") NEAR/1 intervention*) ) ) NOT (SILOID==("PPRN")) |
| 4 | #1 AND #2 AND #3 and Preprint Citation Index (Exclude – Database) |

Appendix Table 6. Global Health Search Strategy

| **#** | **Search Terms** |
| --- | --- |
| 1 | markov processes/ |
| 2 | computer simulation/ |
| 3 | decision making/ or decision analysis/ or decision support systems/ |
| 4 | monte carlo method/ |
| 5 | mathematical models/ or simulation models/ or models/ or econometric models/ |
| 6 | ((decision* adj2 model*) or (decision-analytic* adj2 (model* or approach*)) or ((model* adj3 (approach* or study or simulat*)) and economic) or (simulation adj5 model*) or mathematical model* or (dynamic adj2 model*) or economic model*).tw. |
| 7 | ((markov adj5 (model* or framework* or chain*)) or state transition model*).tw. |
| 8 | ((decision* adj2 tree*) or (decision-making adj2 tree*) or tree model*).tw. |
| 9 | ((micro? adj2 simulation*) or microsimulation or (individual-level adj2 simulation*) or (patient-level adj2 simulation*)).tw. |
| 10 | (monte carlo or discrete event simulation).tw. |
| 11 | 1 or 2 or 3 or 4 or 5 or 6 or 7 or 8 or 9 or 10 |
| 12 | economics/ or socioeconomics/ or welfare economics/ |
| 13 | exp costs/ |
| 14 | cost analysis/ or "cost benefit analysis"/ or "cost control"/ or "cost effectiveness analysis"/ |
| 15 | resource allocation/ |
| 16 | ((economic* adj2 (evaluat* or analys* or assess* or model*)) or (cost adj2 analysis) or (cost adj2 evaluation)).tw. |
| 17 | ((cost adj2 effective*) or (cost-effectiveness adj2 (analys* or study or evaluat* or model* or decision*)) or CEA or ICER or (incremental cost adj2 effectiveness ratio)).tw. |
| 18 | ((cost adj2 utilit*) or (cost-utility adj2 (analys* or ratio* or impact)) or CUA or (QALY or QALYs or "quality-adjusted life year*")).tw. |
| 19 | ((cost adj2 benefit*) or (cost-benefit adj2 (analys* or study)) or (benefit adj2 cost adj2 evaluat*) or CBA).tw. |
| 20 | (economic feasib* or financial viabilit*).tw. |
| 21 | (((value adj2 money) or budget* or costly or costing or price* or expenditure* or (DALY or DALYs or "disability-adjusted life year*")).tw. or health resource.mp.) adj2 (allocat* or utiliz*).tw. |
| 22 | 12 or 13 or 14 or 15 or 16 or 17 or 18 or 19 or 20 or 21 |
| 23 | exp community health workers/ |
| 24 | ("community health worker*" or "community-based health worker*" or "village health worker*" or "community health volunteer*" or "community health agent*" or "community care worker*" or "primary health care worker*").tw. |
| 25 | ((lay adj2 worker*) or ("volunteer health worker*" or "community volunteer*" or "outreach worker*" or "link worker*" or "linkworker*" or "health extension worker*" or "health promoter*" or "community health promoter*" or "community-based practitioner*" or "community case management worker*" or "close-to-community provider*" or "expert patient*" or "home health aide*" or "health auxiliar*" or "paraprofessional*" or "barefoot doctor*" or "frontline health worker*")).tw. |
| 26 | (((community or village) adj3 (health worker* or provider*)) or ((lay or volunteer* or outreach or support or link*) adj5 (worker* or aide* or provider* or practitioner*))).tw. |
| 27 | ((CHW or "community health worker") adj2 intervention*).tw. |
| 28 | 23 or 24 or 25 or 26 or 27 |
| 29 | 11 and 22 and 28 |

## Appendix 2. Data Extraction Form

The data extraction form, developed in Microsoft Excel, was used to systematically record relevant information from each included study.

| **#** | **Author** | **Year** | **Country/region** | **Economic Classification** | **Title** | **Study Objective** | **CHW Target Population** | **Terms used for CHWs** | **Health condition/disease category** |
| --- | --- | --- | --- | --- | --- | --- | --- | --- | --- |
|  |  |  |  |  |  |  |  |  |  |
|  |  |  |  |  |  |  |  |  |  |

| **Decision-analytic Model Type** | **Type of Economic Evaluation** | **Perspective of Analysis** | **Time Horizon** | **Model Structure Described? (Y/N)** | **Model Validation** | | **Sensitivity Analysis** | | | **Assumptions Reported** |
| --- | --- | --- | --- | --- | --- | --- | --- | --- | --- | --- |
|  |  |  |  |  | **Internal** | **External** | **deterministic** | **probabilistic** | **scenario** |  |
|  |  |  |  |  |  |  |  |  |  |  |
|  |  |  |  |  |  |  |  |  |  |  |

| **Outcomes & Effectiveness Data Sources** | **Source of Cost Data** | **Authors' Conclusions** | **Limitations** | **Suggestion for Future Research** |
| --- | --- | --- | --- | --- |
|  |  |  |  |  |
|  |  |  |  |  |

## Appendix 3. Characteristics and Economic Findings of Included Studies

| **Author, year** | **Country/**  **region** | **Economic Classification** | **Health condition/**  **disease category** | **CHW Target Population** | **Type of Economic Evaluation** | **Comparator** | **Outcomes** | **Cost-effective? (Yes/No/Unclear)** |
| --- | --- | --- | --- | --- | --- | --- | --- | --- |
| Tampi et al., 2024^28^ | Tanzania | LOWER-MIDDLE INCOME ECONOMIES | Nutrition and acute conditions | villages with high weekly malaria incidence rates | CEA | Usual care | ICER: US$172.82 /DALY, US$7.74/case averted, US$17901.36/death averted | Y |
| Angell et al., 2021^29^ | Indonesia (rural area of Malang district, East Java province) | UPPER-MIDDLE-INCOME ECONOMIES | MNCH | individuals at high risk of CVD | CUA | Usual care | ICER: US$5242.10/DALY | Y |
| Assebe et al., 2021^30^ | Ethiopia | Ethiopia is currently in a temporary status of unclassification for FY26. | MNCH | pregnant women and mothers, newborns and under-five children, general population | CEA | Without Health Extension Program coverage | ICER: US$94.62/LYG | Y |
| Bone et al., 2021^31^ | India, Pakistan and Mozambique | LOWER-MIDDLE INCOME ECONOMIES, Mozambique is LOW-INCOME ECONOMIES | Communicable diseases | pregnant women (15–49 years in India and Pakistan, and 12–49 years in Mozambique) | CEA | Usual care | Incremental cost per pregnancy: India US$16.15, Pakistan US$14.68, Mozambique US$16.92; High-contact subgroup ICERs: India US$55.24, Mozambique US$62.13, Pakistan US$11.61, per YLL averted | Unclear (Context-dependent) |
| Cho et al., 2023^32^ | Ghana | LOWER-MIDDLE INCOME ECONOMIES | MNCH; Communicable diseases | children aged <5 years | CEA & CBA | Usual care | ICER: US$1467.03/DALY (median), US$2255.40/DALY (mean). | Y |
| Dahal et al., 2025^33^ | Nepal | LOWER-MIDDLE INCOME ECONOMIES | NCDs | aged 30–70 years who are clinically diagnosed with T2DM and that have the ability to respond to a health behaviour intervention | CUA | Usual care | ICER:  Healthcare system perspective: US$674.32 (5y), US$718.85 (10y), US$675.38 (20y), US$670.08 (30y) per QALY gained; Societal perspective: US$762.32 (5y), US$812.16 (10y), US$698.71 (20y), US$759.14 (30y) per QALY gained. | Unclear (Context-dependent) |
| Duan et al., 2021^34^ | Mexico (rural areas, Chiapas) | UPPER-MIDDLE-INCOME ECONOMIES | MNCH | adults with diabetes | CUA | Usual care | ICER: US$3802.79/QALY (lifetime), US$13323.16/QALY (over the first 5 years) | Y |
| Jo et al., 2021^35^ | Bangladesh | LOWER-MIDDLE INCOME ECONOMIES | MNCH | pregnant women | CEA | Usual care | ICER: US$564.80/DALY, US$17089.40/death averted | Y |
| Kumar et al., 2021^36^ | Kenya | LOWER-MIDDLE INCOME ECONOMIES | NCDs | Pregnant women; Newly delivered mothers;  neonates | CEA | Usual care | ICER: US$311.72/DALY | Y |
| Li et al., 2024^37^ | China (rural area) | UPPER-MIDDLE-INCOME ECONOMIES | MNCH; Communicable diseases | hypertensive individuals in rural China | CUA | Usual care | ICER: Dominant (-US$1839.93/QALY) | Y |
| Lubogo et al., 2021^38^ | Uganda | LOW-INCOME ECONOMIES | MNCH; Communicable diseases | children aged <5 years | CEA | Integrated community case management trained-drug seller model | Average Cost-Effectiveness Ratio: US$17.27/appropriately treated U5 patient | Y |
| Mafirakureva et al., 2023^39^ | Cameroon and Uganda | LOW-INCOME ECONOMIES | MNCH; Communicable diseases | children aged <5 years, or those aged 5–14 years and HIV positive | CEA | Usual care | ICER: US$703.41/DALY (Cameroon), US$1100.49/DALY (Uganda) | Y |
| Molanes-Lopez et al., 2024^40^ | Niger (Mayahi district of the Maradi region) | LOW-INCOME ECONOMIES | NCDs | children 6-59 months | CEA | Usual care | ICER: US$117.71/additional SAM case recovered. | Y |
| Mulogo et al., 2024^41^ | Uganda (Bugoye sub-county, Kasese District, rural southwestern Uganda) | LOW-INCOME ECONOMIES | Communicable diseases | children aged <5 years | CEA | Health Facility-based Management | ICER: US$6.82/under five-year child treated | Y |
| Muttalib et al., 2022^42^ | Pakistan (rural areas of Rahimyar Khan) | LOWER-MIDDLE INCOME ECONOMIES | MNCH | pregnant women (specifically those in the third trimester of pregnancy who intended to stay in the study catchment area for at least 1 month after delivery) and their newborn infants | CEA | Usual care | INMB: US$12.27/ iNCK distributed | Y |
| Niyibitegeka et al., 2021^43^ | Burundi | LOW-INCOME ECONOMIES | NCDs | children aged <5 years | CEA | Usual care | ICER: Dominant (provider: -US$1269058.62/DALY; societal: -US$2796484.47/DALY) | Y |
| Reddy et al., 2021^44^ | South Africa (KwaZulu-Natal province) | UPPER-MIDDLE-INCOME ECONOMIES | MNCH; Communicable diseases | general population | CEA | Health-care testing alone | ICER: US$708.57/YLS | Y |
| Bettampadi et al., 2019^45^ | India (Maharashtra) | LOWER-MIDDLE INCOME ECONOMIES | NCDs | children aged <5 years | CEA | Usual care | ICER: US$213.82/DALY | Unclear (Context-dependent) |
| Goudet et al., 2018^46^ | India (Mumbai) | LOWER-MIDDLE INCOME ECONOMIES | Communicable diseases | pregnant women, children younger than 3 years | CUA | Usual care | ICER: US$29.93/DALY | Y |
| Nandi et al., 2016^47^ | India | LOWER-MIDDLE INCOME ECONOMIES | Communicable diseases | newborns | CEA | Baseline of no coverage | Cost savings: US$5847.61 out-of-pocket saved per 1,000 births; Additional savings at 90% coverage: US$812.65. | Y |
| Prinja et al., 2018^48^ | India (Uttar Pradesh) | LOWER-MIDDLE INCOME ECONOMIES | MNCH | pregnant women and newborns | CEA | Usual care | ICER: US$264.13/DALY, US$7556.81/death averted | Unclear (Context-dependent) |
| Rakuomi et al., 2017^49^ | Kenya | LOWER-MIDDLE INCOME ECONOMIES | NCDs | children aged <5 years | CUA | Primary health facility & Tertiary health facility & No access to treatment | ICER: US$6.58/DALY | Y |
| Sharma, 2016^50^ | Kenya | LOWER-MIDDLE INCOME ECONOMIES | MNCH; Communicable diseases | pregnant women and their male partners | CEA | Written invitations for male partners to attend clinic | ICER: US$800.33/DALY | Y |
| Sharma et al., 2018^51^ | western Kenya (Nyanza Province) | LOWER-MIDDLE INCOME ECONOMIES | Cancer | the sexual partners of HIV-positive individuals | CEA | Passive referral & Facility-based testing & Usual care | ICER: US$1084.02/ DALY | Y |
| Gaziano et al., 2014^52^ | South Africa | UPPER-MIDDLE-INCOME ECONOMIES | NCDs | individuals with hypertension and aged 25–74 in South Africa | CEA & CUA | Usual care | ICER: US$431.65/DALY | Y |
| Mash, 2015^53^ | South Africa (underserved communities in Cape Town, Western Cape) | UPPER-MIDDLE-INCOME ECONOMIES | NCDs | people with type II diabetes attending selected community health centers | CUA | Usual care | ICER: US$2423.11/QALY | Y |
| Wagner et al., 2020^54^ | rural South Africa | UPPER-MIDDLE-INCOME ECONOMIES | Health behaviours and social determinants | people with epilepsy | CUA | Usual care | ICER: US$1944.21/QALY (male), US$2416.60/QALY (female) | Y |
| Prezio et al., 2014^55^ | United States | HIGH-INCOME ECONOMIES | NCDs | uninsured predominantly Mexican-American patients with type 2 diabetes mellitus (T2DM). | CUA | Usual care | ICER: US$478.86/QALY | Y |
| Ryabov, 2014^56^ | United States (Hidalgo County, Texas, U.S.-Mexico border) | HIGH-INCOME ECONOMIES | NCDs | individuals with type 2 diabetes who were 30 years of age or older at the beginning of the study. | CEA & CUA | Usual care | ICER: US$19729.62/QALY | Y |
| Smith et al., 2019^57^ | United States | HIGH-INCOME ECONOMIES | MNCH | individuals at risk of cardiovascular disease (CVD) | CUA | Usual care | ICER: Dominant (incremental cost saving US$4607.53 and +0.16 QALYs (52-year-old male), incremental cost saving US$2433.90 and +0.08 QALYs (52-year-old female)) | Y |
| Basu et al., 2017^58^ | USA | HIGH-INCOME ECONOMIES | MNCH | patients with a history of at least 1 ED visit for a chronic condition in the prior year | CBA | Usual care | ED visits averted needed for cost-neutrality (by condition) | Y |
| Mezei et al., 2018^59^ | Uganda | LOW-INCOME ECONOMIES | Nutrition and acute conditions | women at their homes or places of work who were between the ages of 30 and 65, lived and/or worked in Kisenyi, and had access to a mobile phone. | CEA | Clinic-based visual inspection with acetic acid | ICER: US$169.18/YLS, US$312.32/YLS, US$611.63/YLS when performed one, three and five times per lifetime. | Y |
| Drake et al., 2015^60^ | Myanmar | LOWER-MIDDLE INCOME ECONOMIES | Communicable diseases | general population | CEA | ITN interventions & usual care | CER: Accessible areas US$1403.13/YLL, very hard-to-reach US$730.56/YLL; ICER: CHW dominated in most scenarios (in remote areas, CHW+ITN ICER = US$921.25/YLL). | Unclear (Context-dependent) |
| Campos et al., 2020^61^ | El Salvador | UPPER-MIDDLE-INCOME ECONOMIES | NCDs | women aged 30 to 59 years, alone and in concert with clinic-based HPV provider-collection | CEA | No screening & Clinic-based HPV provider-collection alone | ICER: US$1514.79/YLS | Y |
| Hunchangsith et al., 2012^62^ | Thailand | UPPER-MIDDLE-INCOME ECONOMIES | Communicable diseases | HIV-negative TB patients who were sputum smear-positive and were aged 15 years or more | CEA | Self-administered treatment | ICER: Dominant (cost savings:US$4.62 million, health gains: 13,000 DALYs averted) | Y |
| Visram et al., 2020^63^ | UK | HIGH-INCOME ECONOMIES | Cancer | the 30% most deprived communities in County Durham, plus high-need groups including veterans, socially isolated older adults, people with mild to moderate mental health issues, manual workers, and LGBT individuals. | CUA & SROI | No intervention | Cost per QALY gained: US$7404.83; Cost-benefit ratio: US$6.55. | Unclear (Context-dependent) |
| Kolesar et al., 2017^64^ | Madagascar, Ethiopia, and Malawi. | LOW-INCOME ECONOMIES,  Ethiopia is currently in a temporary status of unclassification for FY26 | MNCH; Communicable diseases | rural women of reproductive age; infants and neonates are considered as a subgroup | CEA | Usual care | ICER:  Cost per life saved: Madagascar US$4306.91; Ethiopia US$5533.20; Malawi US$2288.57; Cost per DALY averted: Madagascar US$67.61; Ethiopia US$89.36; Malawi US$40.85. | Y |

Note: Text in quotation marks within the ‘CHW Target Population’ column is reproduced verbatim from the original articles; unquoted text represents reviewers’ summaries. Abbreviations: CEA = Cost-Effectiveness Analysis; CUA = Cost-Utility Analysis; CBA = Cost-Benefit Analysis; CCA = Cost-Consequence Analysis; SROI = Social Return on Investment

## Appendix 4. Data synthesis

Appendix Table 7. Reporting of Model Assumptions, Validation, and Sensitivity Analysis Across Included Studies by Model Type

| **Decision-analytic Model Type** | **Assumptions Reported (Y/N)** | | **Model Validation (Y/N)** | | | | **Sensitivity Analysis (Y/N)** | |
| --- | --- | --- | --- | --- | --- | --- | --- | --- |
|  | **Y** | **N** | **Y(I)** | **Y(E)** | **Y(B)** | **N** | **Y** | **N** |
| **Decision tree / Markov** | | | | | | | | |
| Decision Tree | 11 | 1 | / | / | / | 12 | 12 | / |
| Markov Model | 11 | / | 2 | / | 1 | 8 | 11 | / |
|  | | | | | | | | |
| **Microsimulation / Dynamic Models** | | | | | | | | |
| Markov Microsimulation Model | 2 | / | 1 | / | 1 | / | 1 | 1 |
| Dynamic Transmission Microsimulation | 2 | / | / | 1 | / | 1 | 2 | / |
| Individual-Based Microsimulation | 2 | / | 1 | 1 | / | / | 2 | / |
| Mathematical Microsimulation Model | 1 | / | / | / | 1 | / | 1 | / |
| Microsimulation Model | / | 1 | 1 | / | / | / | 1 | / |
| Dynamic Transmission Model | 1 | / | 1 | / | / | / | 1 | / |
|  | | | | | | | | |
| **Custom / Hybrid / Other** | | | | | | | | |
| DAN | / | 1 | / | / | / | 1 | 1 | / |
| Ready Reckoner | 1 | / | / | / | / | 1 | 1 | / |
| Markov model and Decision Tree | 1 | / | / | / | 1 | / | 1 | / |
| LiST | 1 | / | / | / | / | 1 | 1 | / |
| LiST/FamPlan/TIME | 1 | / | / | / | / | 1 | 1 | / |
|  | | | | | | | | |
| **Total** | **34** | **3** | **6** | **2** | **4** | **25** | **36** | **1** |
| **% (n/37)** | **92%** | **8%** | **16%** | **5%** | **11%** | **68%** | **97%** | **3%** |

Note: the values indicate the number of studies included (n = 37) that reported each element. The percentages in the bottom row represent the proportion of the total number of included studies. '/' denotes not reported. Model validation types are denoted as follows: Y(I) = internal validation; Y(E) = external validation; Y(B) = both; and N = not reported.

Appendix Table 8. Sensitivity Analysis Methods by Decision-Analytic Model Type in CHW Economic Evaluations

| **Decision-analytic Model Type** | **Deterministic Sensitivity Analysis** | | **Scenario Analysis** | **Probabilistic Sensitivity Analysis (PSA)** | **Multi-parameter Sensitivity Analysis** |
| --- | --- | --- | --- | --- | --- |
|  | **One-way Sensitivity Analysis** | **Multi-way Sensitivity Analysis** |  |  |  |
| **Decision tree / Markov** | | | | | |
| Decision Tree | 6 | 1 | 4 | 8 | 5 |
| Markov Model | 8 | 1 | 2 | 7 | 3 |
|  | | | | | |
| **Microsimulation / Dynamic Models** | | | | | |
| Markov Microsimulation Model | 1 | 1 | 1 |  |  |
| Dynamic Transmission Microsimulation | 1 |  | 1 |  |  |
| Individual-Based Microsimulation | 1 | 1 | 2 |  |  |
| Mathematical Microsimulation Model |  |  | 1 |  |  |
| Microsimulation Model |  |  | 1 | 1 |  |
| Dynamic Transmission Model | 1 |  | 1 |  |  |
|  | | | | | |
| **Custom / Hybrid / Other** | | | | | |
| DAN |  |  |  | 1 | 1 |
| Ready Reckoner |  |  | 1 |  |  |
| Markov model and Decision Tree | 1 |  | 1 | 1 |  |
| LiST | 1 |  |  |  |  |
| LiST/FamPlan/TIME | 1 | 1 |  | 1 |  |
|  | | | | | |
| **Total** | **21** | **5** | **15** | **19** | **9** |
| **% (n/37)** | **57%** | **14%** | **41%** | **51%** | **24%** |

Appendix Table 9. Core Methodological Limitations by Decision-Analytic Model Type

| **DAMs / Core Methodological Limitations** | **Data Limitations** | **Model Assumptions and Structural Limitations** | **Methodological Uncertainty** | **Uncertainty Not Fully Accounted** | **Model Dependency on Calibration** | **Effectiveness and Measurement Limitations** | **Study Design Limitations** |
| --- | --- | --- | --- | --- | --- | --- | --- |
| **Decision tree / Markov** | | | | | | | |
| Decision Tree | 5 | 6 | 3 |  |  | 3 | 1 |
| Markov Model | 7 | 5 | 4 | 1 |  |  | 3 |
| **Microsimulation / Dynamic Models** | | | | | | | |
| Markov Microsimulation Model | 1 | 2 |  |  |  |  |  |
| Dynamic Transmission Microsimulation | 1 | 2 | 1 | 1 | 1 | 1 | 1 |
| Individual-Based Microsimulation | 1 | 2 | 1 | 1 |  |  | 1 |
| Mathematical Microsimulation Model |  |  |  |  |  |  |  |
| Microsimulation Model | 1 |  |  |  |  |  | 1 |
| Dynamic Transmission Model |  | 1 |  |  |  | 1 |  |
| **Custom / Hybrid / Other** | | | | | | | |
| Custom Model (DAN / Ready Reckoner) | 1 | 1 | 2 |  |  |  | 2 |
| Markov model and Decision Tree | 1 | 1 |  |  |  |  |  |
| Lives Saved Tool (LiST) | 1 | 1 |  |  |  |  |  |
| Lives Saved Tool (LiST) / FamPlan / TIME-TB Model | 1 | 1 | 1 |  |  | 1 |  |
| **Total** | **20** | **22** | **12** | **3** | **1** | **5** | **10** |
| **% (n/37)** | **54%** | **59%** | **32%** | **8%** | **3%** | **14%** | **27%** |

Note: the number in each cell indicates the number of studies that reported that limitation. The percentages at the bottom of the table represent the proportion of the total number of included studies. Empty cells indicate that no studies of that model type reported the corresponding limitation. As studies could report more than one type of methodological limitation, the categories are not mutually exclusive and the percentages do not sum to 100%.
